# Algorithm for diagnosis of primary vasculitides

**DOI:** 10.1590/1677-5449.009218

**Published:** 2019-03-19

**Authors:** Alexandre Sacchetti Bezerra, Afonso César Polimanti, Rafael Vilhena de Carvalho Fürst, João Antônio Corrêa

**Affiliations:** 1 Faculdade de Medicina do ABC, Disciplina de Angiologia e Cirurgia Vascular, Santo André, SP, Brasil.

**Keywords:** algorithms, classification, diagnosis, differential, systemic vasculitis, vasculitis, algoritmos, classificação, diagnóstico diferencial, vasculite sistêmica, vasculite

## Abstract

Primary vasculitides are diseases with a wide variety of anatomical, clinical, radiological, and laboratory presentations. Primary vasculitides are difficult to diagnose because of the complexity of clinical presentation, which may lead to delayed treatment and increased financial costs of workup investigations involving non-essential tests. Our objective in the present study is to create an algorithm that helps diagnosis of Primary vasculitides. The algorithm presented in this article allows fast, simple and cost-effective diagnosis of primary vasculitides using just clinical concepts and a few laboratory tests.

## Resumo

As vasculites primárias são doenças que possuem apresentações anatômicas, clínicas, radiológicas e laboratoriais muito distintas. Em virtude da complexidade dos quadros clínicos apresentados, as vasculites primárias são de difícil diagnóstico, o que pode promover um retardo no início da terapêutica, além de aumentar os custos financeiros da investigação propedêutica com exames não essenciais. O objetivo deste estudo foi criar um algoritmo que auxilie o diagnóstico das vasculites primárias. O algoritmo apresentado neste artigo permite a realização de diagnóstico rápido, simples e de baixo custo nas vasculites primárias, com a utilização de alguns conceitos clínicos e poucos exames laboratoriais.

## INTRODUCTION

 Vasculitides can be defined as conditions in which an inflammatory process is evident in the vessel wall, associated with target organ damage. 1 Their annual incidence has been increasing over the years. 2

 To date, the literature on the subject does not provide a consensus on the best way to define, classify, and diagnose vasculitis. 2 ^,^ 3

 Vasculitides are classified as primary vasculitides (PV) or secondary vasculitides (SV). 2

 Secondary vasculitides are caused by other diseases, which are considered their etiologies. Thus, a multitude of diseases may induce secondary vasculitis. Cancer, trauma, systemic lupus erythematosus, rheumatoid arthritis, and rocky mountain spotted fever are some of the many examples of diseases that can trigger secondary vasculitis. 2 ^,^ 4

 Primary vasculitides are characterized by immunological modulation failure, 5 with no definite etiology, and patients usually present with consumptive syndrome. 2

 The objective of this study was to create an algorithm to aid diagnosis of primary vasculitides. 

 The classification recommended by the American College of Rheumatology, published in 1994 and revised in 2012 in the Chapel Hill Consensus (CHC2012), organizes Primary and secondary vasculitis based on the size of the vessel involved. 2 ^,^ 6 The scientific relevance of these consensuses has standardized articles on the subject. 

 In order to facilitate diagnostic investigation of primary vasculitides, we also grouped diseases based on vessel caliber, as shown in Table 1 . 

**Table 1:** Names of vasculitides classified by vessel caliber.

**Vessel caliber**	**Disease**
Large vessel vasculitis (> 150 mm)	Takayasu arteritis
	Giant cell arteritis
Medium vessel vasculitis (50-150 mm)	Polyarteritis nodosa
	Kawasaki disease
Small vessel vasculitis (<50 mm)	Granulomatosis with polyangiitis (Wegener’s)
	Microscopic polyangiitis
	Eosinophilic Granulomatosis with Polyangiitis (Churg-Strauss)
	IgA vasculitis (Henoch-Schönlein)
	Cryoglobulinemic vasculitis I, II and III
	Cutaneous vasculitis
	Behçet’s disease
	Leukocytoclastic vasculitis

mm = millimeters.

 It is vital that the physician look for the signs and symptoms already established by CHC2012 when conducting diagnostic screening of primary vasculitides of large and medium vessels. 1 ^,^ 2 ^,^ 6

Table 2 lists the main clinical characteristics of primary vasculitides of large and medium vessels. 

**Table 2:** Names and definitions of vasculitis of large and medium caliber vessels.

**Vessel caliber**	**Disease**	**CHC2012 definition**
Large vessel vasculitis (> 150 mm)	Takayasu arteritis	Arteritis, often granulomatous, predominantly affecting the aorta and/or its major branches. Onset usually in patients younger than 50 years.
	Giant cell arteritis	Arteritis, often granulomatous, usually affecting the aorta and/or its major branches, with a predilection for the branches of the carotid and vertebral arteries. Often involves the temporal artery. Onset usually in patients older than 50 years and often associated with polymyalgia rheumatica.
Medium vessel vasculitis (50-150 mm)	Polyarteritis nodosa	Necrotizing arteritis of medium or small arteries without glomerulonephritis or vasculitis in arterioles, capillaries, or venules, and not associated with antineutrophil cytoplasmic antibodies (ANCAs).
	Kawasaki disease	Arteritis associated with the mucocutaneous lymph node syndrome and predominantly affecting medium and small arteries. Coronary arteries are often involved. Aorta and large arteries may be involved. Usually occurs in infants and young children.

mm = millimeters.

**Figure 1:**
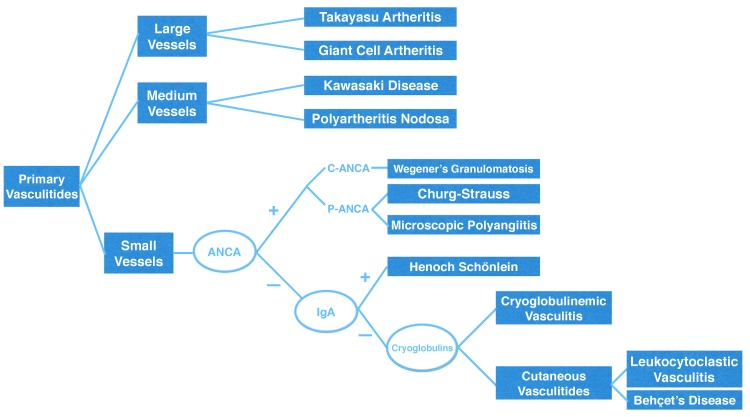
Diagnostic algorithm for primary vasculitides.

 Our algorithm, shown in Figure 1 , does not recommend laboratory tests in the diagnostic routine for primary vasculitides of large and medium vessels, since the clinical characteristics of the four diseases involved are sufficient. 7 ^-^ 11

 For primary vasculitis of small vessels, we advise judicious and economical test ordering. 

 In addition to benefiting the patient, wise diagnostic investigation hastens specific therapy and is less of a burden on health services. Tables 3 and 4 list costs of commonly ordered tests for diagnostic investigation of vasculitis in the United States and on the Brazilian Public Healthcare Service, respectively. 

**Table 3:** Costs of some tests for primary vasculitis diagnostic screening in United States.

**Exams**	**Average cost in US dollars**
Complement; antigen; each component	10.50
CH50	19.39
Duplex scan of lower extremity arteries	143.39
IgM	10.50
Percutaneous transluminal coronary angiography	337.22
IgG	10.50
Transcranial Doppler study	157.43
Myeloperoxidase (MPO)	18.91
Antistreptolysin O	5.20
Proteinase 3 (PR3)	18.91
Antinuclear antibodies	5.20
Rheumatoid factor	4.88
IgA	10.50
Echocardiography	211.40
Cryoglobulins	5.48
Esophagus, gastroesophageal reflux test	389.18
Sedimentation rate	2.00
C reactive protein	4.10
HIV test	15.17
Nerve conduction studies	189.42
Duplex scan of extracranial arteries	144.46
Hepatitis B serology	40.40
Hepatitis C antibody	19.13
Intravascular Doppler velocity	220.45
Blood count; leukocyte (WBC); automated	3.13

Data based on April 2018 on NYS Medicaid Laboratory Fees Schedule 12 .

**Table 4:** Costs of some tests for primary vasculitis diagnostic screening in Brazil.

**Exams**	**Average Cost in US Dollars**
Complement; antigen; each component	4.49
CH50	2.42
Duplex scan of lower extremity arteries	10.36
IgM	4.49
Percutaneous transluminal coronary angiography	160.92
IgG	4.85
Transcranial Doppler study	30.62
Myeloperoxidase (MPO)	*
Antistreptolysin O	0.74
Proteinase 3 (PR3)	*
Antinuclear antibodies	4.49
Rheumatoid factor	1.07
IgA	4.85
Echocardiography	10.45
Cryoglobulins	0.60
Esophagus, gastroesophageal reflux test	35.43
Sedimentation rate	0.71
C reactive protein	0.74
HIV test	22.25
Nerve conduction studies	7.06
Hepatitis B serology	4.85
Hepatitis C antibody	4.85
Intravascular Doppler velocity	*
Blood count; leukocyte (WBC); automated	0.71

Data based on November 2018 on DATASUS (SIGTAP) 13 . American Dollar exchange rate on 24, November, 2018: US$1.00 = R$3.82 14 .

* No financial reference in DATASUS 13 .

 It is a myth that diagnosis can be speeded up in cases of primary vasculitis of small vessels that are suitable for non-noble tissue biopsies. 3

 Since biopsy is expensive, difficult to perform and inconclusive in a large number of patients with small vessel vasculitis, we recommend a quick and staged sequence of laboratory tests ( Figure 1 ). 

## DISCUSSION

 Initially, we recommend a laboratory test to measure anti-neutrophil cytoplasmic antibodies (ANCA). 7

 These antibodies are immunoglobulins against azurophil granules containing many different proteins, such as myeloperoxidase (MPO), elastase, proteinase 3 (PR3), and cathepsin G, among others. 7 ^,^ 15

 Antibodies that target myeloperoxidase are called P-ANCA, since they usually present with a perinuclear staining pattern in indirect immunofluorescence. If the antibodies target Proteinase 3, they are known as C-ANCA, because they exhibit a central cytoplasmic staining pattern. 7 ^,^ 15

 Radice et al. strongly confirm the accuracy of C-ANCA (PR3-ANCA) and P-ANCA (MPO-ANCA) for diagnostic investigation of vasculitis. 16 ^,^ 17

 Wegener's granulomatosis or granulomatosis with poliangiitis (WG/GPA) is characterized by granulomatous necrotizing vasculitis and lung-kidney syndrome. A positive C-ANCA result has sensitivity and specificity exceeding 90% for active WG/GPA disease. 18

 Primary vasculitis with lung-kidney syndrome may occur with negative C-ANCA and positive P-ANCA. This form is classified as microscopic polyangiitis. 2 ^,^ 17 Previously established algorithms using P-ANCA for microscopic polyangiitis (MPA) diagnosis have high accuracy. 

 Lionaki et al. 17 conducted a 22-year cohort study with 502 patients in which 81% of patients with microscopic polyangiitis were positive for P-ANCA. 

 Primary vasculitides of small vessels without lung-kidney syndrome can also occur with negative C-ANCA and positive P-ANCA. This form is known as eosinophilic granulomatosis with polyangiitis or Churg-Strauss syndrome (EGPA/CSS). These patients may have asthma, eosinophilia, and peripheral neuropathy. 3 ^,^ 6

 When the results of P-ANCA and C-ANCA are negative in diagnostic investigation of primary vasculitides of small vessels, a second stage of laboratory tests is needed, assaying immunoglobulin A ( Figure 1 ). 

 In a retrospective study with 417 patients with IgA vasculitis or Henoch-Schönlein purpura (HSP) treated at a single center over 37 years, Calvo-Rio et al. did not detect positive ANCA in any patients. 19

 Patients with HSP may have anaphylactoid purpura, abdominal pains, and peripheral neuropathy. 20

 When the results of P-ANCA, C-ANCA and IgA are all negative, it is necessary to conduct a third stage of laboratory tests, for cryoglobulin I, II and III ( Figure 1 ). 

 Cryoglobulins are insoluble immunoglobulins at temperatures below 37 degrees Celsius. These deposits generate inflammatory processes in innumerable tissues. 4 ^,^ 21

 Systemic cryoglobulinaemic vasculitis (SCV) type I is characterized by monoclonal IgM and is associated with lymphoproliferative diseases, such as myeloma and lymphoma. 22 Type II SCV is characterized by monoclonal and polyclonal IgM and IgG and is linked with infectious diseases, such as hepatitis C. 22 ^,^ 23 Type III SCV is characterized by polyclonal IgM and IgG and is associated with inflammatory diseases such as ulcerative colitis and Crohn’s disease. 3 ^,^ 24

 When the results of P-ANCA, C-ANCA, IgA and cryoglobulins are negative in diagnostic investigation of primary vasculitis of small vessels, a fourth and final step is needed. 

 The final phase is to investigate the possibility of leukocytoclastic vasculitis and Behçet's disease. These cutaneous vasculitides (CV) also affect small vessels ( Figure 1 ). 25

 Leukocytoclastic vasculitis or hypersensitivity vasculitis, is a vasculitis of small vessels that mainly affects venules through deposition of immune complexes and has a pathognomonic microscopic profile. Histopathology shows visible karyorrhexis with inflammatory infiltrate, fibrinoid necrosis, and neutrophil nucleus fragmentation in the vascular wall. 3 ^,^ 26 ^,^ 27

 Behçet's disease is a systemic vasculitis of both arteries and veins, described with recurrent genital and oral ulcers, uveitis, and ectropion. Inflammatory lesions in the central nervous system and large vessels are rarely found. 28 Its most common presentation affects mainly small vessels. 2 ^,^ 28

 Diagnosis of cutaneous vasculitides is effective and accessible because of their unique clinical, epidemiological, and histopatological features. 25

 At most health services, the routine diagnostic algorithm of investigation for primary vasculitides is extremely complex, laborious, time-consuming and expensive. 

 The diagnostic sequence shown above is not intended to eliminate additional clinical, radiological, or laboratory workup, which may be essential in selected cases. However, arriving at a diagnosis in a labor-saving and affordable manner is imperative. 

 In conclusion, the algorithm presented in this article enables fast, simple, and cost-effective diagnosis of primary vasculitides using just clinical concepts and a few laboratory tests. 
